# Immunostimulating Commensal Bacteria and Their Potential Use as Therapeutics

**DOI:** 10.3390/ijms242115644

**Published:** 2023-10-27

**Authors:** Bonita McCuaig, Yoshiyuki Goto

**Affiliations:** 1 Project for Host-Microbial Interactions in Symbiosis and Pathogenesis, Division of Molecular Immunology, Medical Mycology Research Center, Chiba University, Chiba 260-8673, Japan; 2 Division of Pandemic and Post-Disaster Infectious Diseases, Research Institute of Disaster Medicine, Chiba University, Chiba 260-8673, Japan; 3 Division of Infectious Disease Vaccine R&D, Research Institute of Disaster Medicine, Chiba University, Chiba 260-8673, Japan; 4 Chiba University Synergy Institute for Futuristic Mucosal Vaccine Research and Development (cSIMVa), Chiba University, Chiba 260-8673, Japan

**Keywords:** gut microbiome, colonization resistance, microbiome host crosstalk, SCFA, bacteriocins, immunoactive, microbiome intervention, fecal transfer, probiotic, prebiotic, postbiotic

## Abstract

The gut microbiome is intimately intertwined with the host immune system, having effects on the systemic immune system. Dysbiosis of the gut microbiome has been linked not only to gastrointestinal disorders but also conditions of the skin, lungs, and brain. Commensal bacteria can affect the immune status of the host through a stimulation of the innate immune system, training of the adaptive immune system, and competitive exclusion of pathogens. Commensal bacteria improve immune response through the production of immunomodulating compounds such as microbe-associated molecular patterns (MAMPs), short-chain fatty acids (SCFAs), and secondary bile acids. The microbiome, especially when in dysbiosis, is plastic and can be manipulated through the introduction of beneficial bacteria or the adjustment of nutrients to stimulate the expansion of beneficial taxa. The complex nature of the gastrointestinal tract (GIT) ecosystem complicates the use of these methods, as similar treatments have various results in individuals with different residential microbiomes and differential health statuses. A more complete understanding of the interaction between commensal species, host genetics, and the host immune system is needed for effective microbiome interventions to be developed and implemented in a clinical setting.

## 1. Introduction

The human body is host to many diverse microbes, with the largest community inhabiting the gastrointestinal tract (GIT). There has recently been an increased interest in how these communities interact with the human host and affect its overall health. These microbial communities consist of viruses, archaea, eukaryotes (fungi, protists, arthropods, etc.), and bacteria. The bacterial members of this community are the most well studied and will be the focus of this review. Strains of commensal bacteria have often coevolved alongside a specific taxonomic group of hosts, sometimes being specific to a single species [1,2]. An extreme example are the “infant type” *Bifidobacterium* spp., which have co-evolved alongside humans and specialize in utilizing carbohydrates found in human breastmilk, allowing extensive colonization only during nursing [3,4]. The microbiome is also specific to each area of the host GIT with the community changing along its length [5,6]. Many studies to date have focused on the fecal microbiome because it is the most accessible for sampling. The bacterial community present in the feces is most like that in the colon; however, it is not a good representative of the bacterial communities in more proximal areas of the GIT [7]. It is generally accepted that the fetus is sterile within the womb and colonization of the GIT begins during birth [8,9], although some have argued that microbiome colonization begins in utero [10]. The GIT microbiome changes rapidly during early life and stabilizes as we age. The neonatal intestine is aerobic and initial colonization is by facultative anaerobes belonging to the phyla Actinomycetota and Bacillota (formerly Actinobacter and Firmicutes); as the intestine matures and becomes anaerobic, Pseudomonadota and Bacteroidota (formerly Proteobacteria and Bacteriodetes) become more prevalent [4,11]. This succession pattern to a healthy adult microbiome has long term implications for host health, and disruptions of the GIT community early in life can affect vaccine response and increase the chances of autoimmune, neurological, lung, and gastrointestinal disorders later in life [11,12,13,14,15,16,17].

The advent of “omic” studies has made identifying and studying the large number of unculturable bacteria within the human GIT possible. Metagenomic studies allow us to identify the bacteria within the community with more accuracy than the use of marker genes (16S rRNA being the most commonly used). With the improvements in sequencing and data analysis, it has become clear that marker genes often underestimate the diversity of the community [18]. The use of metagenomic sequencing also allows for the prediction of the metabolic pathways present, giving insight into the metabolic potential of the community [19]. Large scale collaborations have created datasets of human genome information paired with microbiome information. Differences in sampling, sample preparation, sequencing technology, and species identification are challenges that must be addressed when utilizing data from multiple studies [20]. Despite these challenges, researchers have been able to identify a link between Bifidobacterium species and specific genotypes related to the *LCT* gene [21,22,23]. A link between ABO blood groups and multiple bacteria taxa has also been identified [21,22]. Identifying genetic variation with links to the microbiome is challenging. There are usually confounding social, diet, and environmental factors, which also affect the microbiome. Further studies with consistent methods in diverse populations are needed to untangle these factors [20]. With this burst of available information, researchers have been able to identify specific changes in the GIT microbiome of people suffering from many diseases and disorders, as well as possible genetic links to disease susceptibility.

**Figure 1:**
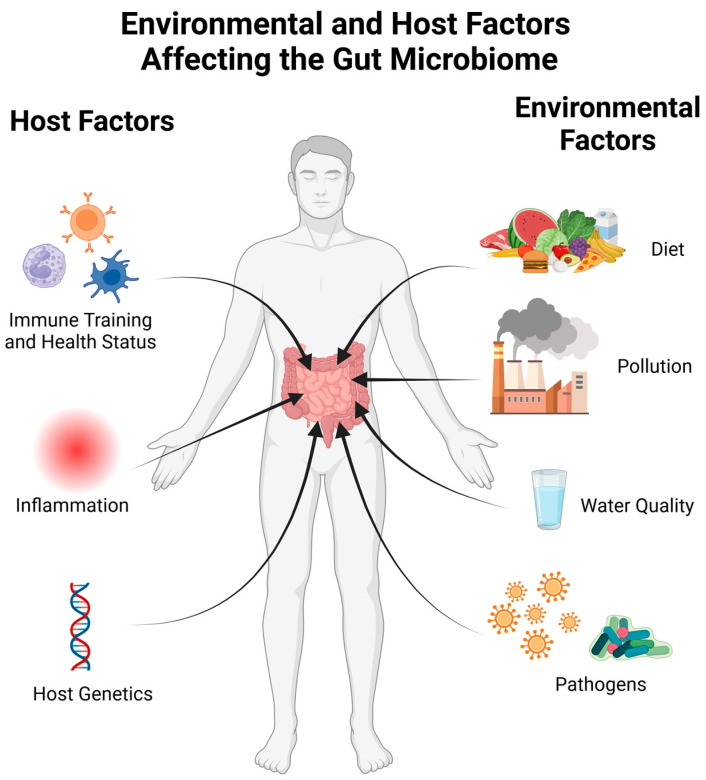
A visualization of factors influencing the microbiome. The human host affects the microbiome through their current health status as well as immune training from previous infections or vaccinations. Both systemic and gut inflammation can perturb the microbiome, and a number of host genetic factors have been linked to the gut microbiome composition. Environmental factors such as diet, water quality, pollution, and exposure to pathogens also have consequences within the gut microbiome. Created with BioRender.com (accessed on 23 October 2023).

The gut microbiome is also susceptible to perturbation by a number of environmental factors. Exposure to environmental pollutants from air, water, or food contamination affects the gut microbiome [24,25,26]. Air pollution can cause damage and inflammation in the lungs. This activation of the innate immune system can have systemic affects and cause changes to the intestinal microbiome [25,27,28]. A study in Dutch individuals also showed a link between smoking—past and present, as well as secondhand smoke—and the gut microbiome [29]. The contamination of water with heavy metals and other oxidizers disrupts the redox balance within the intestinal environment. This can directly affect bacteria sensitive to oxidative stress, such as Faecalibacterium, or cause damage to intestinal tissues, resulting in a disruptive immune response [24,26]. Bisphenol A (BPA) is a common contaminant known to affect the human endocrine system. Exposure to this chemical has been linked to decreases in *Bifidobacteria* and *Akkermansia* levels [25]. The factors affecting the microbiome can be thought of as originating from the host or the host’s environment, as highlighted in Figure 1.

Healthy adults with an intact microbiome are unlikely to be colonized by a number of pathogens; however, the disruption of the microbiome allows for colonization by these pathogens. *Clostridioides difficile* pathogenesis often occurs after prolonged antibiotic treatment has disrupted the microbiome; however, it is present in most healthy adults with no adverse effects. The reconstitution of the microbiome has become an approved medical intervention for prolonged *C. difficile* cases [30,31]. *Vibrio cholera* is able to colonize infant mice with undeveloped immune systems, causing a disease state, but adult mice cannot be colonized without first disrupting the GIT microbiome [32,33]. Similarly, it has been proposed that cholera is only able to colonize and cause disease in humans when the microbiome is disrupted, often by malnutrition or diarrhea of an unrelated source [34,35]. *Listeria monocytogens* is an agent of food borne illnesses that is also better able to infect mice when the microbiome is disturbed; resistance has been linked to products of the microbiome such as SCFAs and antimicrobials [36]. The microbiome also provides protection against *Salmonella* colonization, recently reviewed in Rogers et al. [37]. A healthy microbiome provides protection against intestinal pathogens through direct competition as well as the priming and training of the host immune system. A more in-depth discussion of how the microbiome restricts pathogen growth, the bacterial products involved, which commensal bacteria produce them, and interventions to harness this protection are discussed below. With this review, we hope to present a comprehensive look at how the gut microbiome affects human health and present possible modes of action for potential therapeutic approaches.

## 2. Mechanisms by Which the Microbiome Provides Colonization Resistance

Studies in gnotobiotic models have shown that in the absence of commensal bacteria, the host immune system fails to develop properly, with little innate immune response and a poor response of the adaptive immune system when exposed to bacteria. Commensal organisms are needed to train the host immune system and improve their resistance to pathogens [16,17,38]. This immune training has implications throughout the host body and is not localized to the GIT. The microbiome improves pathogen resistance through nutrient and oxygen sequestering, the production of antimicrobial compounds, occupying attachment sites, the stimulation of mucus production, the tightening of tight junctions, the regulation of inflammation, and the training of innate and adaptive immunity [39,40,41,42,43,44]. Colonization resistance is often the result of immunoactive compounds produced by the bacterial community. These include microbe-associated molecular patterns (MAMPs), extracellular vesicles (EVs), microbe-derived anti-inflammatory compounds, the degradation of harmful compounds, and beneficial microbial metabolic products that stimulate the immune system, increasing its effectiveness against pathogens [38,43,45,46].

The microbiome provides colonization resistance through environmental engineering, the production of antibacterial compounds, and direct competition for electron acceptors, nutrients, and physical space within the intestine [37,47]. Some commensal bacteria produce antimicrobial compounds. These compounds reduce the competition from other species and allow the commensal to establish itself within the intestine [48,49]. These compounds are discussed in more detail in the Section 3. Production of organic acids lowers the pH of the intestinal lumen and changes the redox potential of the environment, which can interfere with virulence factors [37]. Of particular interest are short-chain fatty acids (SCFAs) [37,50]. Increased oxidative stress in the intestinal lumen has been linked to changes in the microbiome that lead to dysbiosis and proliferation of pathogens [51,52]. Conversely, oxygen sequestering by commensal bacteria can limit the growth of pathogens. This is the proposed mode of action for the colonization resistance provided by resident *Enterobacterales* against virulent *Salmonella* sp. [37]. The residential bacteria utilize the available nutrients, and an established microbiome is resistant to the introduction of new species [37,53,54]. This is true for pathogens but also the introduction of beneficial bacteria, and it is a challenge for microbiome interventions involving probiotics. Finally, commensal bacteria can occupy attachment sites that are required for pathogen virulence. Segmented filamentous bacteria (SFB) attach to intestinal epithelial cells in a similar manner to *Salmonella*, effectively blocking *Salmonella* from attaching to the intestinal epithelium and preventing disease [2,55,56]. Similarly, non-infectious *E. coli* can occupy attachment sites used by virulent *E. coli* strains [53]. Commensal bacteria that are closely related to pathogens are often able to occupy the ecological niche preferred by the pathogen and, once established, are effective in excluding them from the intestine [37,53,54]. The innate immune system in the GIT is constitutively active, as it is always stimulated by commensal bacteria. A healthy gut has effective barrier function and appropriate inflammation responses. An overly sensitive inflammation response can lead to a loss in barrier function, referred to as a “leaky gut”, allowing metabolites, proteins, and even bacteria to cross the epithelium, causing sepsis. This can lead to poor health outcomes throughout the body, and has been linked to intestinal, metabolic, lung, skin, and neurological disorders [57,58,59]. However, changes to the microbiome associated with these conditions are not consistent, and it is unclear if the microbiome changes that are observed are a cause or effect of the condition in humans, although fecal transplantation can transfer these disorders to recipient mice [59,60,61].

The adaptive immune system is also very active within the GIT. Antigens in the lumen are constantly sampled, especially in the specialized areas of the Peyer’s Patches, which are located along the small intestine, most densely in the ileum. The development of Peyer’s Patches is reduced in germ-free mice [62]. They are areas with a thinner mucus coating, no villi, and specialized M-cells that allow dendrites to extend into the lumen and sample antigens, including food, viruses, and bacteria. Located below the Peyer’s Patch is an area of lymph tissue where antigen-presenting cells can interact with naïve B-cells, triggering the subsequent production of sIgA in the lamina propria [63]. Sampling through the Peyer’s Patch is a source of undiversified IgA in the intestine [64], although luminal sampling near goblet cells has also been observed [65,66]. The differentiation of T-cells is also directly affected by the microbiome. CD4^+^ naïve T-cells differentiate into regulatory T-cells (Treg) or Th17 cells in the presence of TGF-β, with Th17 cells requiring the additional presence of IL-6. Beneficial commensal bacteria lower the production of IL-6, promoting the differentiation into Treg cells, which produce IL-10 and reduce inflammation levels [67,68]. Antigen sampling within the gut is likely to stimulate immunotolerance because of regulatory cytokine ratios present in the healthy intestine. This propensity for tolerance is extended to other areas of the body, as the immune cells trained in the GIT migrate to other parts of the body [69]. Peyer’s Patches are also the most prominent areas for T-cell dependent class switching, while T-cell independent class switching takes place within the laminal propria [17,63]. Immune training through luminal sampling in the GIT trains the adaptive immune system and affects the systemic immune response.

## 3. Beneficial Bacterial Products

Beneficial bacterial products are diverse in nature, and some are structural compounds of the microbial cell (LPS, peptidoglycan, unmethylated DNA, etc.), while others are products of microbial metabolism (microbial anti-inflammatory molecule, SCFA, bacteriocins, etc.). These bacterial products are involved in crosstalk with the host. In many experiments and clinical trials, the exact compounds responsible for the benefit to the host is not clear, and the mechanism of action is often expounded by in vitro experiments involving cell lines or in animal models.

The innate immune system is stimulated by MAMPs such as peptidoglycans, unmethylated CpG DNA, LPS, etc. These patterns are recognized by pattern recognition receptors (PRRs) that can be attached to the cell membrane within the periplasm or secreted outside the cell. Membrane-bound PRRs include Toll-like receptors (TLRs), C-type lectin receptors (CLRs), and Nod-like receptors (NLRs), which are found on a variety of cells within the GIT and throughout the body [44]. Peroxisome proliferator-activated receptors (PPARs) are nuclear receptors that regulate inflammatory and metabolic processes and are found in cells throughout the body [70]. The innate immune system, including the epithelium barrier, is the first line of defense against pathogens and, until recently, was believed to have little or no specificity and no immune memory, providing a rapid and consistent response to all potential pathogens [43,71,72,73,74]. Recent work has shown that the innate immune system does respond differently to repeated challenges. Vaccination with live attenuated *Bacillus Calmette-Guerin* provides non-specific protection against multiple pathogens [40,71], possibly from changes to glucose metabolism by immune cells [72] and the reprogramming of stem cells within the germinal centers of the bone [73]. By increasing innate immune cell production and cytokine production, the innate immune system may have more immune memory than previously thought. While MAMPs are non-specific, the presence of non-pathogenic commensals can provide some cross protection against similar pathogens.

Extracellular vesicles (EVs) are spherical membranous vesicles that can contain molecules such as proteins and nucleic acids [45,70,74] as well as the MAMPs associated with cell wall components. EVs are produced by both prokaryotic and eukaryotic cells and are utilized for intercellular communication [75]. EVs produced by *Faecalibacterium prausnitzii* [70,74], *Akkermansia muciniphila* [76], and *Bacteroides thetaiotaomicron* [45] have all been shown to interact with TLR and PPARs, affecting cytokine production. *B. thetaiotaomicron* were shown to interact with TLR1,2,3, and 7 to help immune cell survival, reducing inflammatory signals [45]. EVs from *A. muciniphila* induced the phosphorylation of AMPK, a tight junction protein activator. This was supported by reduced intestinal permeability in mice colonized by *A. muciniphila* [76]. The production of IL-10 was increased by *F. prausnitzii* and *A. muciniphila* EVs through reduction in the NF-kB inhibitor, NFKBIL1 [74,76]. Commensal EVs also reduce the expression of inflammatory cytokines such as IL-1, IL-2, IL-6, IL-12, and IFN-γ [70,77].

SCFAs are the product of the bacterial fermentation of indigestible fiber in the large intestine. The most common SCFAs are acetate, propionate, and butyrate [47,78,79], although less abundant SCFAs can also influence the immune system [80]. SCFAs interact with G protein-coupled receptors (GPR) 41 and 43; these receptors are found in intestinal epithelial cells but also peripheral organs and blood cells, affecting inflammation throughout the body [81,82]. Butyrate has been reported to reduce inflammation throughout the body, reducing the production of IL-1β, IL-2, IL-12, IFN-γ, and TNF-α, while upregulating the production of IL-10 [47,83]. Butyrate also promotes the differentiation of Treg cells, which increases tolerogenic responses [67,84]. Butyrate can be directly used by colonocytes as an energy source, promoting a healthy intestinal epithelium. Healthy colonocytes promote barrier function by increasing mucus production, promoting healthy tight junctions and the increased production of antimicrobial proteins in Paneth cells [47,80,81,82]. SCFAs also lower the pH of the intestinal environment, inhibiting the growth of many pathogens [37,85]. Propionate was shown to limit the growth of *Salmonella enterica* serovar Typhimurium in vitro through changes to the intercellular pH [86]. Butyrate is able to reduce the growth of multiple *C. difficile* strains [87] and *L. monocytogens* [50] in culture, showing an inhibition of this pathogen unrelated to host immune response [78].

## 4. Beneficial Bacteria

The benefit of fermented foods such as yogurt, cheese, kefir, and natto to gut health has been known for centuries [88]. Many probiotics that are currently on the market are species that were historically used in food production, particularly lactic acid-producing bacteria (LAB). LAB include bacteria belonging to the genera *Lactobacillus*, *Lactococcus*, and *Bifidobacterium* and are commonly found in fermented milk products. LAB are well studied, and their probiotic benefits and mechanisms of action have been recently reviewed in detail by Tiwari and Tiwari (2022) [48]. LAB produce bacteriocins, bacteriocin-like molecules, hydrogen peroxide, and carbon dioxide, which restrict the growth of the major pathogens *Listeria* [49], *Clostridium*, and *Salmonella* [89]. LAB also interact with the immune system, influencing immunotolerance and generally promoting Treg differentiation as well as reducing inflammation responses within the intestine [48,78,89,90]. The use of LAB in food production has allowed for a large-scale production of probiotics for commercial sale with relatively little regulatory opposition.

A healthy GIT microbiome is hard to define because of the high variation seen across populations [91,92,93]. Generally, a high alpha diversity is indicative of a healthy microbial community. A loss of diversity and a low evenness score are commonly seen in the microbiomes of people suffering from dysbiosis and many medical conditions [16,92,94]. The most abundant bacterial phyla in the mammalian GIT are Bacillota and Bacteroidota; these two phyla constitute ~80% of a healthy microbiome. These abundances are often compared and referred to as the older nomenclature of Firmicutes:Bacteroidetes or the F:B ratio. A large F:B ratio is often an indication of a healthy gut microbiome, while a low F:B ratio often indicates dysbiosis [95,96]; however, an increased F:B ratio has been linked to obesity [96,97,98]. The age of the host is a major factor when using this ratio and must be taken into consideration [16,94,99,100]. Some Bacillota are oxygen tolerant and more abundant in the GIT of neonatal and young hosts, with the oxygen sensitive Bacteroidota being a secondary colonizer [6,14,101].

**Figure 2:**
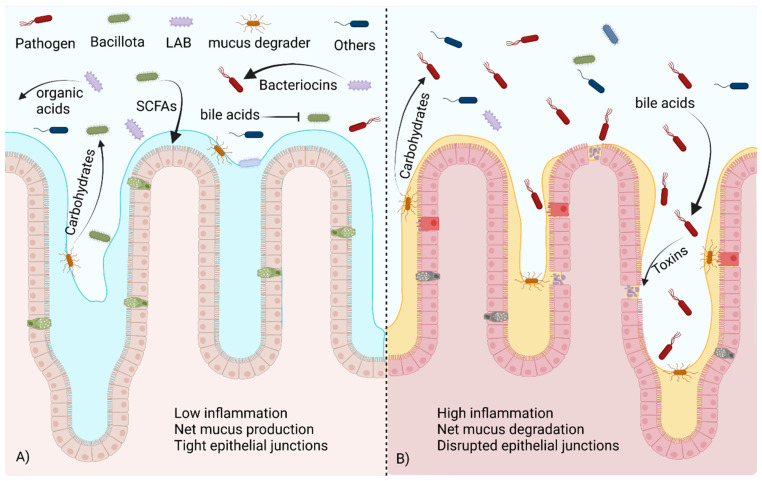
A healthy commensal bacteria population versus one in dysbiosis. (**A**) In the healthy gut microbiome, beneficial Bacillota metabolize bile acids and utilize carbohydrates freed by mucus degraders to produce SCFAs, promoting mucus production and healthy tight junctions in the epithelium. LABs produce organic acids and bacteriocins that control pathogen populations. (**B**) In dysbiosis, the presence of unmodified bile acids stimulates toxin production via pathogens. Without competition from beneficial species, pathogens are able to utilize carbohydrates released by mucus degraders to expand their population, damaging the epithelium. Created with BioRender.com (accessed on 23 October 2023).

The majority of bacteria within the phylum Bacillota are gram-positive, spore-forming bacteria with various levels of oxygen tolerance. This phylum was identified as beneficial to gut health early in the study of the GIT microbiome. Bacillota are often involved with the fermentation of fiber in the colon. The main products of interest in this fermentation are SCFAs. These products of bacterial fermentation can be used as an energy source by colonocytes, and they can enter the bloodstream and react with cell receptors located in distant organs [75,83,85,102,103]. The diversity found within the phyla Bacillota likely plays a role in the inconsistent results when this high-level classification is considered. While total Bacillota present is often correlated to SCFA production/levels [96], this is not always the case. Strategies such as this rely on the accurate identification and prior knowledge of fermentation abilities, both of which may be lacking, resulting in inaccuracies. To complicate matters further, there are a number of pathogenic species within the Bacillota, the most common of which are *Clostridiodes difficile* and species belonging to the genus *Staphylococcus.* While high-level generalizations are appealing, it is an oversimplification of the complex nature of the gut ecosystem, and identification to a more specific classification. A visualization of many of these concepts can be seen in Figure 2. Below, we discuss specific groups that are thought to be beneficial to host health and immune function.

The genus Faecalibacterium contains commensal bacteria that are also known to ferment indigestible fiber into SCFAs [75]. Faecalibacterium are obligate anaerobes and are unable to colonize the human GIT early in life but become common after 2–3 years, becoming ~5% of the microbiome in adults [104]. *F. prausnitzii* produces a protein that reduces inflammatory signals in mammalian cells [105]. This has been shown to be beneficial in Crohn’s disease and IBS [105,106]. Increased oxidative stress is associated with gut inflammation, and Faecalibacterium has been suggested as a potential marker species because of its relatively high abundance and oxygen sensitivity [107,108]. An increase in oxidative stress during inflammation has been suggested as one cause of dysbiosis [109]. Many SCFA producers are sensitive to oxygenation stress, while opportunistic pathogens tend to have a higher oxidative stress tolerance. Members of the phylum Verrucomicrobiota, such as *Akkermansia muciniphila*, also produce SCFAs but have a higher oxygen tolerance [83,110]. This phylum is most abundant early in life but is present in most adult microbiomes [94,101]. *Akkermansia* spp. and other members of the Verrucomicrobiota digest the mucus lining the GIT as a nutrient source, releasing metabolites and freeing carbohydrates that can be further degraded by other members of the community [83]. *A. muciniphila* and *Bifidobacterium* sp. have been considered for use as a probiotic because of their ability to promote the growth of other beneficial bacteria [85,111], Figure 2A. It can be considered an environmental engineer as it can colonize intestines with oxidative stress, reduce the oxygen levels through reduced inflammation and metabolism, and produce nutrients for the more oxygen sensitive SCFA producing bacteria allowing them to colonize further improving gut health [76,83,112]. Species from the genus *Blautia*, particularly *Blautia obeum*, have been shown to provide protection from a *Vibrio cholera* infection [113]. The presence of bile acids induces the production of toxin-coregulated pilus (TCP) and type VI secretion system (T6SS) proteins in *V. cholera*. *Blautia* species reduce primary bile acids into secondary bile acids, downregulating these virulence factors [114,115], as shown in Figure 2B. However, bile acids are toxic to *L. monocytogenes*, and the degradation of these products may lead to a higher susceptibility to listeria infection [36]. Commensal bacteria can also mimic quorum-sensing signals essential for virulence in cholera colonization. The most well documented is the production of autoinducer 2 (AI2) by *B. obeum*, which blocks production of TCP, reducing virulence within the intestine [33,34].

Segmented filamentous bacteria (SFB) have both a unique structure and life cycle, discussed in some detail in Hedblom et al., 2018 [2]. These bacteria attach directly to the intestinal epithelium, most often in the ileum, causing changes to the host cell and resulting in a pedestal-like structure at the site of attachment and a loss of microvilli in the immediate area. The preference of attachment site varies by species and includes enterocytes, M-cells, goblet cells, and junctions between cells in the ileum. SFB are often host specific [56], possibly through the recognition of differences within the flagellar proteins [116]. The attachment method of SFB is similar to *Salmonella* sp., blocking attachment sites from the pathogen [2]. Despite this similarity in attachment methods, SFB do not incite an inflammatory response; however, they do elicit an immune response.

**Figure 3:**
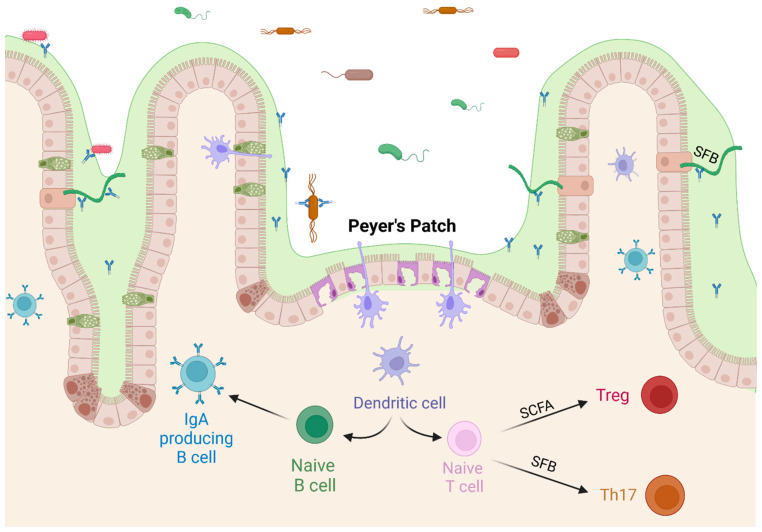
Commensal bacteria affect T cell differentiation. SCFAs produced by commensal bacteria upregulate the differentiation of regulatory T cells, and SFB stimulate differentiation of Th17 cells, both pathogenic and regulatory. Created with BioRender.com (accessed on 23 October 2023).

SFB have been shown to induce immunological changes in mice, and the abundance of SFB correlates with vaccine response in a number of studies. The relationship between SFB and immune response has been extensively investigated in mice. Ivanov and colleagues (2009) showed that the presence of SFB induces Th17 cell differentiation through Toll Like Receptor 5 (TLR5) signaling [117]. SFB also induce the production of serum amyloid A (SAA), IL-17, and IL-22, which stimulate TH17 cell differentiation [55,117,118]. This increased antimicrobial defense was sufficient to protect against *Citrobacter rodentium* [117]. TH17 cells can be subdivided into those that clear pathogens from mucosal surfaces and those that promote immunotolerance [119,120]; the relative abundance of these cells is dependent on cytokine concentrations with pathogenic TH17 cells being induced by IL-23 exposure [68]. The overproduction of TH17 cells has been implicated in autoimmune conditions, including those centered in the GIT such as Crohn’s disease, irritable bowel syndrome (IBS), and others [118,119]. However, the negative effects of TH17 upregulation can be mitigated by the upregulation of anti-inflammatory cytokines by butyrate production in the GIT [47], depicted in Figure 3.

## 5. Therapeutic Interventions to Improve the GIT Microbiome

As we discussed, commensal microbes and their effects of “colonization resistance” contribute to preventing host diseases. Therefore, commensal bacteria as a therapeutic intervention have garnered attention in the medical world with the rise of antibiotic resistant bacteria and the push to reduce antibiotic use. A better understanding of the mechanism of action of the microbiome in immune response and pathogen resistance does suggest that there is potential for both the treatment and prevention of microbiome-related disorders. However, the complex ecology of the GIT means that individuals will have unique reactions to microbiome intervention. Bacterial products will elicit different responses dependent on the bacterial community present, the preexisting inflammation levels, and the immune state and genetics of the host. Because of these factors, microbiome interventions may have to be personalized to each patient, requiring pretreatment investigations into the microbial community present to identify proper treatment options. However, there is less concern about this when the microbiome has been previously decimated by antibiotics, radiation, or chemotherapy.

**Figure 4:**
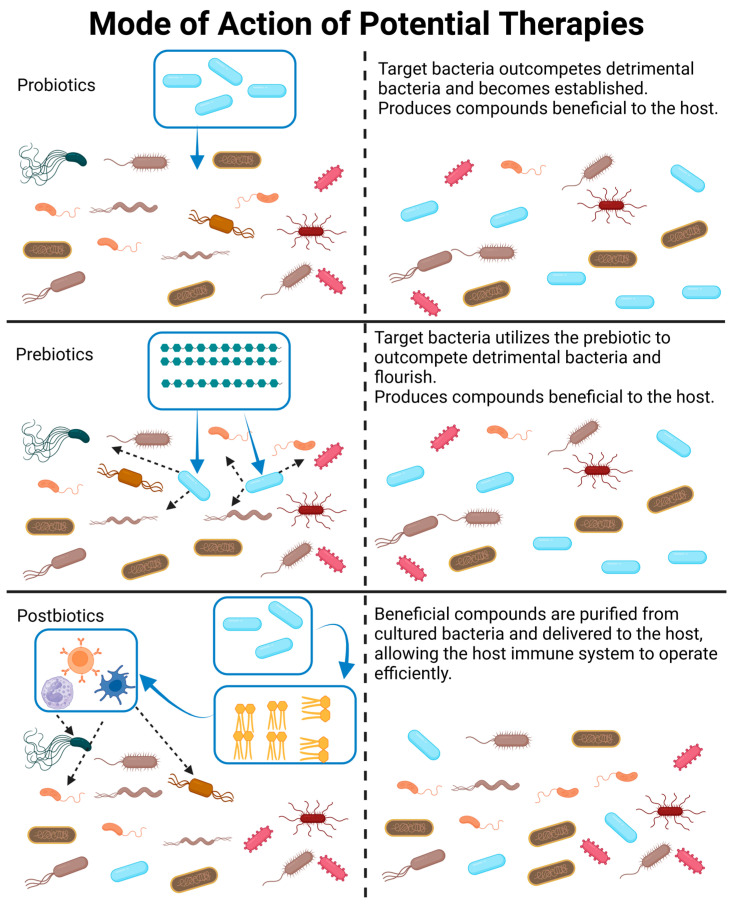
A visual representation and description of how probiotics, prebiotics, and postbiotics modify the gut microbiome. Please note that probiotics may be a combination of bacterial species, which is not depicted in this figure. Likewise, postbiotics may be a combination of bacterial products or whole killed bacteria delivered to the GIT. Created with BioRender.com (accessed on 23 October 2023).

Therapeutic interventions can broadly fall into four categories: (1) the introduction of bacterial communities via fecal transplantation (FT) or the introduction of a predetermined bacterial consortia, (2) probiotics, (3) postbiotics, and (4) prebiotics. Categories 1 and 2 involve the introduction of live organisms, which can be a major concern. It is difficult to receive government approval for the development of new probiotics due to concerns regarding safety and quality control. This difficulty has led to the search for non-living alternatives, such as prebiotics, which stimulate the expansion of existing beneficial species, or postbiotics, which are non-living bacterial products. Potential uses and concerns with each of these categories will be discussed in the sections below and are visualized in Figure 4.

### 5.1. Fecal Transplantation and Bacterial Consortiums

FT has been effective in treating chronic *Clostridioides difficile* infections when antibiotics have been ineffective [30,31]. FT can be conducted through oral administration, enema, or placed directly into the small intestine via colonoscopy or transendoscopic enteral tubing [121]. When administering FT through the oral route, it is important to protect the bacterial community from the acidic environment of the stomach, while the enema route places the community within the colon and does not immediately seed bacteria into the small intestine. An advantage of FT is that a functional community with intact interspecies relationships is transplanted [121,122]. Identifying appropriate donors is challenging, and the microbiome changes throughout the host’s lifetime, meaning previously successful donors may no longer be suitable [123]. The largest downfall of FT is that opportunistic pathogens are present in all fecal samples, even from healthy donors. The current state of FT as a clinical treatment has been reviewed in Gupta et al., 2022 [31]; Biazzo and Deidda, 2022 [121]; and Mahmoudi and Hossainpour, 2022 [122]. The adult immune system is trained and primed by the host’s native GIT microbiome. Some studies have suggested that FT is most successful when the donor has a similar genetic background and diet to the recipient [124]. As host genetics and diet are two major factors affecting the microbiome, selecting donors based on these factors will increase the similarity between the donor microbiome and the recipient’s natural microbiome, increasing the likelihood of successful recolonization [124]. In the case of patients undergoing treatments for a condition unrelated to the GIT that is likely to disrupt the microbiome (i.e., prolonged antibiotic use, chemotherapy, etc.), it is possible to store fecal samples pretreatment, recolonizing the patient with their own native microflora [125]. There is also the possibility of the creation of autoprobiotics, where beneficial bacteria from fecal samples are cultured to probiotic levels and then reintroduced to the host to boost beneficial bacteria levels [126].

An alternative to FT is the production of bacterial consortia. This eliminates the need to find healthy donors, and potential pathogens can be excluded while still introducing beneficial bacteria with healthy interactions, which is a major concern in immunocompromised patients [126,127,128]. The development and maintenance of these consortia is a major hurdle in their production and has similar constraints to probiotics, as discussed below. Many gut bacteria are difficult to culture, as they require stable temperatures, anaerobic or microaerobic conditions, and molecular cross feeding to flourish.

### 5.2. Probiotics

The health benefits of fermented foods have been known for centuries, and probiotics are often delivered through fermented foods such as yogurt, cheese, natto, etc. [88,127,129]. The ideal probiotic is easily cultured, stored, and delivered to the patients. It must also be possible to deliver the living bacteria to the proper area of the intestine for it to colonize, which means surviving passage through the stomach into the intestines. The effectiveness of the probiotic is also dependent on its ability to colonize despite competition by resident bacteria. The genera *Lactobacillus* and *Bifidobacter* have many advantages as probiotics [126,130]. They are found in many fermented foods and known to be safe for human consumption. They are also acid resistant, and a small population can survive past the stomach to colonize the intestine. Finally, they are LAB and provide all the previously discussed benefits [89,131,132].

Species from the genus *Bacillus*, such as *B. subtilus* and *B. ceres*, are currently sold as probiotics for both livestock and humans [133,134,135,136]. These bacteria are capable of sporulation and have been shown to survive passage through the stomach in livestock [137,138]. In livestock, bacilli probiotics improve immune function, increase barrier function within the intestines, and reduce oxidation stress within the intestine [133,139,140]. The ability of *Bacillus* sp. to colonize the intestine long term is under debate, and the levels within the feces are significantly reduced once oral ingestion is stopped [141].

### 5.3. Postbiotics

Postbiotics can include purified bacterial products or whole killed bacteria. Postbiotics avoid the concerns of administering live organisms to patients, but the benefits are temporary and are lost when treatment is concluded [90,142,143]. Often inactivated bacteria are administered with the culture medium, providing any beneficial compounds produced alongside the MAMPs present in the killed bacterial cells, having similar effects on the host immune response [144]. Purified EVs have also been shown to have similar effects as live cell cultures on Caco-2 cells in a culture [45,70]. Bacteriocins have been purified from a number of species and used in food preservation and livestock studies [145,146,147]. However, some compounds can be altered via passage through the stomach or absorbed in the small intestine before reaching the colon, reducing effectiveness, and the compounds may need protective preprocessing. For example, SCFAs can be esterified to ensure delivery to the colon where their effects are most beneficial [47]. Further work is needed to identify purification methods and the effects of postbiotics on human health.

### 5.4. Prebiotics

Prebiotics are compounds that cannot be digested by the host but are accessible to the microbiome. Often the goal of prebiotics is to stimulate the production of beneficial products, but they may also be chosen to simply expand the population of beneficial bacteria. The most common prebiotics are indigestible fibers. These compounds are undigested by the host and reach the lower intestine intact, where they can be fermented by commensal bacteria, often producing SCFAs as a result [51,85,148]. Pascale et al., 2022 [148] recently reviewed the use of pectins as a prebiotic. They found that pectins promoted SCFA production, particularly acetate. The conversion of acetate to propionate and butyrate varied, likely influenced by the resident microbiome community [75]. Fructooligosaccharides are another common prebiotic. Costa et al., 2022, discuss the effects of this prebiotic on inflammation and gut immune response [51]. The increased intake of non-digestible starches increased the SCFA production of test subjects on a controlled diet [85]. As previously discussed, SCFAs are directly beneficial to the host; by promoting the growth of beneficial SCFA-producing bacteria, the growth of potential pathogens is also restricted through competitive exclusion and colonization resistance.

One challenge of prebiotics is that they can only promote the growth of bacteria that are present in the microbiome. This can be addressed by administering probiotics alongside prebiotics, often referred to as synbiotics. By providing the nutrients preferred by the probiotic, they are given an advantage over other bacteria within the GIT. The administration of a commercial synbiotic alongside *Bifidobacterium longum* significantly reduced mucosal inflammation compared to the probiotic alone [149]. Similar results were observed when a fiber synbiotic was combined with a five species *Bacillus* spp. bacterial consortium [150]. Identifying appropriate prebiotic and probiotic combinations could improve the efficacy of such treatments in a clinical setting.

In some cases, the restriction of bacterial nutrients is a more beneficial course of action. This requires dietary changes rather than the addition of a prebiotic. Diets low in fermentable oligosaccharides, disaccharides, monosaccharides, and polyols (FODMAP) have been suggested to treat inflammatory bowel disorders. The microbial community is changed by removing the resources. It is the act of restricting the nutrient that causes the change [151]. However, the efficacy of this treatment is debated [152], and the suitability of this treatment may rely on the pre-existing microbial community [153].

## 6. Future Direction

While microbiome interventions are a promising area of study, there is still much to be learned in how they interact with the resident microbiome and the host. Introduction of a new species to a complex ecosystem can be challenging, as they are often unable to establish themselves within the preexisting community. It is also difficult to predict the effects this will have on the ecosystem and, consequentially, the host. A more complete knowledge of the interactions between species within the GIT, such as cross feeding and the production of antimicrobial compounds, will improve our ability to accurately predict the effects of microbiome interventions. Similarly, a better understanding of which species can utilize prebiotic compounds will help avoid the unintended proliferation of potential pathogens.

Microbial interventions have shown the most consistent results when the resident population has been disrupted, most often by medical interventions such as antibiotic use or chemotherapy. In these cases, the GIT is more easily colonized by the desired bacteria, and there are less unpredicted consequences due to interactions with the resident bacteria. But, even in disorder, the established microbiome is highly variable, and the circumstances of each individual patient must be taken into consideration when planning microbiome interventions. Ideally, each patient would have a fecal sample sequenced to identify the established microbiome and a customized plan created; however, this is unrealistic. Microbial interventions could be semi tailored through the identification of pre-existing conditions and a general understanding of the patient’s diet, allowing assumptions of the resident microbiome to be made.

## 7. Conclusions

The GIT microbiome influences the host immune system and can have serious effects on the systemic immune response and the overall health of the host. Recent work has highlighted the connection between the microbiome and intestinal, metabolic, cardiovascular, respiratory, and neurological conditions. While the GIT microbiome is a complex ecosystem, we can influence the composition of the community using fecal transplants, probiotics, prebiotics, and postbiotics. However, the same treatment may have opposing effects in different individuals because of the variable nature of the resident bacteria, the immune status of the host, and the nutrients available to the microbiome. Therefore, it is important that further research is conducted to improve our understanding of the interspecies relationships and the consequences of microbiome interventions under different conditions. The development of protocols for the creation of disorder-specific or personalized treatments will improve the efficacy of microbiome interventions.

## Data Availability

No new data were created or analyzed in generating this review. Data sharing is not applicable to this article.
